# Dynamic changes of oligomeric amyloid β levels in plasma induced by spiked synthetic Aβ_42_

**DOI:** 10.1186/s13195-017-0310-6

**Published:** 2017-10-17

**Authors:** Seong Soo A. An, Byoung-sub Lee, Ji Sun Yu, Kuntaek Lim, Gwang Je Kim, Ryan Lee, Shinwon Kim, Sungmin Kang, Young Ho Park, Min Jeong Wang, Young Soon Yang, Young Chul Youn, SangYun Kim

**Affiliations:** 1 Department of Bionanotechnology, Gachon University, Incheon, Republic of Korea; 2 Research and Development, PeopleBio Inc., Gyeonggi-do, Republic of Korea; 3 Department of Neurology, Seoul National University College of Medicine & Neurocognitive Behavior Center, Seoul National University Bundang Hospital, 300 Gumidong, Bundang-gu, Seongnam-si, Gyeonggi-do, 463-707 Republic of Korea; 4 Department of Neurology, Veterans Health Service Medical Center, Seoul, Republic of Korea; 5 Department of Neurology, Chung-Ang University College of Medicine, Seoul, Republic of Korea

**Keywords:** Multimer detection system, Alzheimer’s disease, Amyloid-β, Oligomers, Blood biomarker, Synthetic amyloid-β, ELISA, Plasma test

## Abstract

**Background:**

A reliable blood-based assay is required to properly diagnose and monitor Alzheimer’s disease (AD). Many attempts have been made to develop such a diagnostic tool by measuring amyloid-β oligomers (AβOs) in the blood, but none have been successful in terms of method reliability. We present a multimer detection system (MDS), initially developed for the detection of prion oligomers in the blood, to detect AβOs.

**Methods:**

To characterize Aβ in the blood, plasma was spiked with synthetic amyloid-β (Aβ) and incubated over time. Then, the MDS was used to monitor the dynamic changes of AβO levels in the plasma.

**Results:**

Increasing concentrations of AβOs were observed in the plasma of patients with AD but not in the plasma of normal control subjects. The plasma from patients with AD (*n* = 27) was differentiated from that of the age-matched normal control subjects (*n* = 144) with a sensitivity of 83.3% and a specificity of 90.0%.

**Conclusions:**

Synthetic Aβ spiked into the blood plasma of patients with AD, but that of not elderly normal control subjects, induced dynamic changes in the formation of AβOs over time. AβOs were detected by the MDS, which is a useful blood-based assay with high sensitivity and specificity for AD diagnosis.

## Background

Amyloid-β (Aβ) is a major factor in the pathogenesis of Alzheimer’s disease (AD) [1–3]. Aβ may be involved in the cognitive impairment of memory that leads to AD, particularly in the form of aggregated 42-amino acid isoform of the Aβ peptide (Aβ_42_), which is a major neurotoxic species among Aβ isomers, including Aβ_40_, Aβ_42_, and other truncated forms of Aβ [4–10]. Since the initial identification of these Aβ isomers in bodily fluids, the measurement of Aβ levels in the blood and cerebrospinal fluid (CSF) has been a research priority [11, 12]. Three biomarkers in CSF, specifically Aβ_42_, total tau, and hyperphosphorylated tau, are widely accepted as AD determinants on the basis of their close correlations with AD pathology [13, 14]. Diagnostic imaging using positron emission tomography (PET) is the preferred method of investigating pathological and functional changes in the brain of patient with AD. ^11^C- or ^18^F-labeled Pittsburgh compound B-positron emission tomography (PiB-PET), which involves binding to amyloid depositions, is particularly beneficial for understanding the underlying processes of AD. Therefore, this type of imaging is used both in research and in the clinic to differentiate patients with AD from control subjects and individuals with other types of dementia [15–17].

Several noninvasive diagnostics for AD, based on diverse biomarkers in the saliva, urine, and blood, have been reported and are still in the research phase of development [18–21]. Mapstone et al. [22] identified a panel of ten plasma phospholipids as potential diagnostic biomarkers of AD, which included lysophosphatidylcholine, phosphatidylcholine metabolites, and acylcarnitine metabolites. This plasma lipid panel predicted AD conversion, suggesting 90% sensitivity and 85% specificity in differentiating an at-risk group from the cognitively intact group. Despite encouraging results, low positive predictive values limited the clinical usefulness of this panel as a screening tool in subjects aged 70–80 years or younger. In another study, significant differences in soluble CD40 (sCD40) and sCD40 ligand (sCD40L) levels in plasma were observed between AD cases and control subjects. sCD40 was approximately three times higher in patients with AD than in control subjects with sensitivity and specificity of 68% and 84%, respectively. Similarly, concentrations of sCD40L were 2.27 times higher in AD cases than in control subjects with sensitivity and specificity of 51% and 76%, respectively [23]. A biomarker panel of cortisol, pancreatic polypeptide, insulin-like growth factor binding protein 2, β_2_-microglobulin, vascular cell adhesion molecule 1, carcinoembryonic antigen, matrix metalloproteinase 2, CD40, macrophage inflammatory protein 1α, superoxide dismutase, and homocysteine was shown to significantly increase in plasma from patients with AD. In addition, apolipoprotein E (ApoE), epidermal growth factor receptor, hemoglobin, calcium, zinc, interleukin (IL)-17, and albumin were revealed to be decreased in patients with AD. Cross-validated accuracy measures from the Australian Imaging, Biomarker & Lifestyle Flagship Study of Ageing (AIBL) cohort reached a mean (SD) of 85% (3.0%) for sensitivity and specificity and 93% (3.0) for the AUROC. A second validation using the Alzheimer’s Disease Neuroimaging Initiative cohort showed accuracy measures of 80% (3.0%) for sensitivity and specificity and 85% (3.0) for the AUROC [24]. Eighteen signaling blood proteins in hematopoiesis, immune responses, apoptosis, and neuronal supports were suggested to differentiate patients with AD from control subjects with close to 90% accuracy and also to identify patients who would convert from mild cognitive impairment to AD 2–6 years later [25].

Among these, methods employing blood-based biomarkers have been focused on the detection of amyloid-β oligomers (AβOs) and other surrogate biomarkers of AD [26–31]. Experimental cross-sectional analyses undertaken to detect AβOs in the plasma have demonstrated limited and inconclusive results [26–28]. Other blood-based surrogate biomarkers, including ApoE, inflammatory markers (IL-8, IL-1a), Aβ autoantibodies, total serum cholesterol, and microRNAs (specifically miR-9, miR-29a, miR-29b, miR101, miR-125b, miR-132, miR-134, and miR-181c), have also demonstrated variability as disease correlates [29–31]. Although there have been difficulties in developing methods for AD diagnosis using blood-based biomarkers, a reliable and reproducible blood-based assay is still needed for clinical use [32].

A multimer detection system (MDS) was originally developed to detect prion oligomers in the blood of scrapie-infected animals. MDS is a sandwich enzyme-linked immunosorbent assay (ELISA) that preferentially detects oligomers over monomers by creating steric hindrance between capturing and detection antibodies that are specific to a unique/overlapping epitope [33].

In the present study, MDS for AD was developed to detect AβOs using two different antibodies against the N-terminus of Aβ. Initially, MDS was unable to differentiate AβOs in the blood of patients with AD from those of normal control subjects. Synthetic Aβ was then spiked into the plasma of patients with AD and control subjects. Using MDS, the dynamic changes of AβO formation were detected in the spiked plasma of patients with AD but not in the spiked plasma of control subjects. Therefore, in this study, we evaluated the dynamic changes of AβO levels in the plasma of patients with AD compared with those of normal age-matched control subjects.

## Methods

### Clinical data

This study was approved by the institutional review board of Seoul National University Bundang and Chung-Ang University Hospital [B-0905-075-003, B-1202-145-003, C2012048(743), C2013142(1102)]. Pooled plasma samples were collected from 11 patients with AD and 9 elderly normal control subjects, and individual plasma samples of 24 patients with AD and 29 healthy elderly normal control subjects were collected from either Seoul National University Bundang Hospital or Chung-Ang University Hospital (Table 1). Written informed consent was obtained from all patients who participated in this study or from their caregivers. AD cases were each diagnosed with a probable AD amnestic type on the basis of clinical criteria of the National Institute on Aging-Alzheimer’s Association workgroups within a clinical setting with clinical data and follow-up longer than 6 month before inclusion into PiB-PET or CSF studies. Hence, the recruited patients were clinically well-characterized patients with AD, and only they were included in the study. They were diagnosed with AD after initial workup and had not shown any possibility of other neurodegenerative disorders except AD or secondary dementia disorders on the basis of more than 6 months of follow-up. The Mini Mental State Examination, identification of the ApoE phenotype, PET imaging with PiB and ^18^F-fluorodeoxyglucose, and CSF analysis were performed. The characteristics of all participants are described in Table 1. Fifty-one additional plasma samples from elderly normal control subjects were included to avoid false positivity (Table 2).

**Table 1:** Characterization of patients with Alzheimer’s disease and healthy normal control subjects

	AD	Healthy normal control subjects
Total sample number	24	29
Sex		
Female	13 (54.2%)	16 (55.2%)
Male	11 (45.8%)	13 (44.8%)
Age, years (SD)	67.6 (±7.4)	62.4 (±5.7)
Education, years (SD)	13.1 (±3.9)	13.2 (±3.5)
CDR-SOB, mean	6.35	0.03
MMSE score, mean	17.7	29.03
ApoE ε4, %	47.8	21.7
Note test	1	0
Number of plasma samples	24	29
CSF markers	23	28
Aβ_42_, pg/ml, mean (SD)	258.6 (±70.8)	464.8 (±114.4)
p-Tau, pg/ml, mean (SD)	58.6 (±18.6)	28.0 (±14.3)
t-Tau, pg/ml, mean (SD)	132.1 (±61.8)	62.1 (±20.3)
PiB-PET number	23	28
Mean SUVR	1.57	1.14
FDG-PET number	18	28
Mean SUVR	0.9	1.06

*Abbreviations: Aβ*_*42*_ Amyloid-β 1–42 peptide, *AD* Alzheimer’s disease, *ApoE* Apolipoprotein E, *CDR-SOB* Clinical Dementia Rating Sum of Boxes, *CSF* Cerebrospinal fluid, *FDG* ^18^F-fluorodeoxyglucose, *MMSE* Mini Mental State Examination, *PET* Positron emission tomography, *PiB* ^11^C-Pittsburgh compound B, *p-Tau* Phosphorylated tau protein, *SUVR* Standardized uptake value ratio, *t-Tau* Total tau protein

**Table 2:** Supplementary information on healthy normal control subjects

	Healthy normal control subjects
Total sample number	51
Sex	
Female	21 (43.1%)
Male	29 (56.9%)
Age, years (SD)	62.25 (±7.89)
Education, years (SD)	10.35 (±3.29)
CDR-SOB	0.03
MMSE score	28.27

*CDR-SOB* Clinical Dementia Rating Sum of Boxes, *MMSE* Mini Mental State Examination

### Sample preparation

Blood samples were collected in heparin-containing tubes and centrifuged at 850 × *g* for 30 minutes. The plasma (supernatant) was divided into aliquots and stored at −80 °C until analysis. Plasma samples of patients with AD (*n* = 11) and elderly normal control subjects (*n* = 9) were separately pooled for initial method optimization, whereas the remaining samples were assessed individually.

### Preparation of synthetic Aβ_42_

Lyophilized AggreSure β-Amyloid (1–42) peptide (AnaSpec, Fremont, CA, USA) and double-mutant F19S/L34P Aβ_42_ (mutAβ_42_; AnyGen Co., Ltd., Gwangju, South Korea) were each dissolved in 50 mM Tris/150 mM NaCl (pH 7.2) at a concentration of 1 mg/ml and then sonicated for 5 minutes to obtain a homogeneous solution. The peptide solution was further diluted with phosphate-buffered saline containing Tween 20 (PBST; Sigma-Aldrich, St. Louis, MO, USA) to a desired concentration of 10 μg/ml. Solutions of diluted peptides were divided into aliquots and kept at −80 °C until further use.

### Thioflavin T assay

Aβ aggregation was monitored using a thioflavin T (ThT) assay kit following the suggested protocol of the manufacturer (AnaSpec). Ninety microliters of test sample and 10 μl of 2 mM ThT solution were added to each well of a 96-well plate (Thermo Fisher Scientific, Waltham, MA, USA), and the plates were incubated for different lengths of time. Then, changes in ThT fluorescence intensity were detected by measuring excitation and emission wavelengths of 440 nm and 484 nm, respectively, using a multispectrophotometer (Victor 3^TM^; PerkinElmer, Waltham, MA, USA) with 15 seconds of shaking before reading and analysis.

### TEM

AβO, protofibrils, and fibrils were characterized by TEM at various incubation times (0, 1, 3, 6, 24, and 48 h). Five microliters of each sample was applied to carbon-coated TEM grids that had previously been glow-discharged for 3 minutes in the air and immediately negatively stained (~5 seconds) with 2% uranyl acetate. Excess solution was removed with blotting paper. Image acquisition was carried out using a Philips CM10 transmission electron microscope (Philips Research, Eindhoven, The Netherlands) with an accelerating voltage of 80 kV.

### Sodium dodecyl sulfate-PAGE and immunoblotting

The aggregation state of Aβ was also analyzed by sodium dodecyl sulfate-PAGE followed by Western blotting. Synthetic peptide samples were electrophoresed on a 10–20% Tris-Tricine precast gel (Bio-Rad Laboratories, Hercules, CA, USA) and visualized by Coomassie blue staining (Bio-Rad Laboratories). After electrophoresis, the proteins were transferred to a polyvinylidene fluoride membrane (Bio-Rad Laboratories), which was blocked with 2% Block Ace (Bio-Rad Laboratories) in Tris-buffered saline containing Tween 20 (TBST; Sigma-Aldrich) for 1 h at room temperature (RT) under conditions to reduce nonspecific binding. The membrane was incubated for 1 h at RT with a horseradish peroxidase (HRP)-conjugated FF51 antibody (FF51-HRP antibody; PeopleBio Inc., Seoul, South Korea) diluted in 0.4% Block Ace in TBST. Proteins bound to the antibody were visualized with 3,3′,5,5′-tetramethylbenzadine reagent (Sigma-Aldrich).

### MDS for Alzheimer’s disease

A modified MDS was used to measure AβOs. With this method, epitope-overlapping antibodies specific for the N-terminus of Aβ were used to capture and detect the Aβ antigen in its multimeric or oligomeric form. Because MDS was initially developed to detect prion oligomers using prion antibodies, over 100 sets of antibodies against Aβ were screened (data not shown). In addition, in-house Aβ antibodies were developed. The mouse monoclonal antibody 6E10 (BioLegend, San Diego, CA, USA) and an in-house FF51-HRP antibody were chosen to detect AβOs in our modified MDS, owing to their sensitivity and specificity. The epitopes for these antibodies overlap at the N-terminus of Aβ. The FF51 antibody specifically recognizes amino acid residues 1–4 of Aβ.

To use MDS, the 6E10 antibody was coated overnight at 4 °C in the wells of a 96-well black plate (Thermo Fisher Scientific) at a dilution of 3 μg/ml in carbonate-bicarbonate buffer (Sigma-Aldrich). The plates were blocked for 2 h with 0.4% Block Ace (100 μl) at RT. After washing three times with PBS (Sigma-Aldrich), the plate was stored at 4 °C until use. Prior to the assay, aliquots of plasma samples were thawed at 37 °C for 15 minutes. Ten microliters of plasma, 4.04 μl of HBR-1, a HAMA blocker (Scantibodies Laboratory, Santee, CA, USA), and PBST were mixed. We spiked the synthetic Aβ_42_ into plasma mixture and incubated it at 37 °C for the indicated durations.

The plasma sample mixture and serially diluted standards were added to each well of the plate in a total volume of 100 μl. The plates were incubated at RT for 1 h. After washing three times with TBST, the FF51-HRP antibody in TBST containing 0.4% Block Ace was added to the wells, and the plate was incubated for 1 h at RT. To increase the sensitivity of detection, 100 μl/well of enhanced chemiluminescence substrate solution (Rockland Immunochemicals Inc., Limerick, PA, USA) was used, and the luminescent signal was detected and quantified using a Victor 3^TM^ multispectrophotometer.

### Measurement of Aβ monomers (Aβ_40_ and Aβ_42_)

Sandwich ELISAs were performed to measure Aβ_40_ and Aβ_42_ monomer levels. Aβ_40_ monomers were captured with the 11A50 antibody (specific for the C-terminus of Aβ_40_) and detected with the 1E11 antibody conjugated to biotin. Aβ_42_ monomers were captured with the 12F4 antibody (specific for the C-terminus of Aβ_42_) and detected with the 1E11 antibody conjugated to biotin.

### Statistics

Statistical evaluations were performed using the Mann-Whitney *U* test followed by the calculation of two-tailed *p* values to determine the significance between groups.

## Results

### Measuring dynamic changes of AβO levels with MDS

**Fig. 1:**
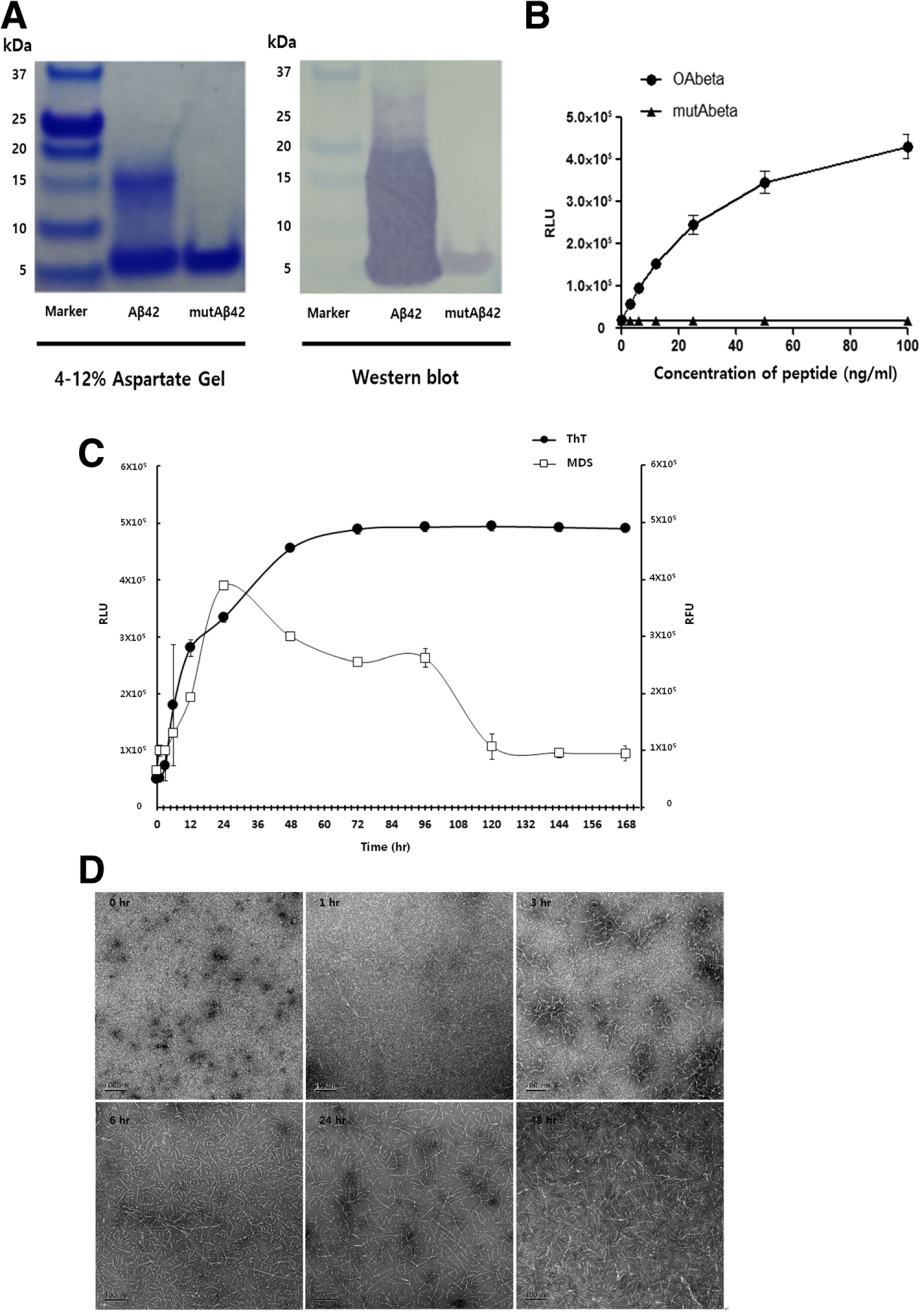
Detection of synthetic amyloid-β 1–42 peptide (Aβ_42_). **a** Freshly dissolved synthetic Aβ_42_ and double-mutant Aβ_42_ (mutAβ_42_) were visualized on the 4–12% aspartate PAGE gel by Coomassie blue staining and on the Western blot by FF51-horseradish peroxidase (HRP) antibody. **b** Standard curve for multimer detection system (MDS). Synthetic peptides were serially diluted and measured by 6E10/FF51-HRP MDS assay. Change of Aβ_42_ over different times (**c** and **d**). Aβ_42_ was incubated at 37 °C at the indicated time points. To measure amyloid-β oligomer (AβO), an MDS assay was used (**c**), and fibrillization of Aβ_42_ over time was measured by thioflavin T (ThT) binding assay (**d**). TEM images over time by Aβ_42_ in buffer vehicle are shown. Data are mean ± SD. *RLU* Relative luminescence units, *RFU* Relative fluorescence units

Aβ_42_ was characterized by gel electrophoresis, Western blotting, and TEM before it was spiked into plasma samples (Fig. 1). On the basis of Coomassie blue staining of dissolved Aβ_42_, a smear band containing monomers and low-molecular-weight oligomers ranging between 4 and 18 kDa in size was detected, as shown in Fig. 1a. A double-mutant, Aβ_42_ (F19S/L34P; mutAβ_42_), was used as a monomeric Aβ control because this mutant has significantly reduced aggregation potential as shown by Western blotting, which yielded a specific band with an approximate molecular weight of 4–5 kDa. The specificities of the wild-type and mutant monomer bands were verified by MDS, as shown in Fig. 1b. MDS was capable of detecting AβOs composed of Aβ_42_ in a concentration-dependent manner employing half serial dilutions from 100 ng to 3.13 ng, whereas no signal was detected when using mutAβ_42_. Thus, MDS specifically recognizes AβOs but not Aβ monomers.

As shown in Fig. 1c, changes in Aβ_42_ oligomer levels over an incubation period of 144 h were monitored using an MDS and the ThT assay. The MDS detected a continual increase in Aβ_42_ oligomer levels from 0 to 24 h after the start of incubation, followed by a decline until 120 h, at which point the levels remained relatively stable. Conversely, the ThT assay showed an increase in Aβ_42_ oligomer levels from 0 to 48 h, at which point changes in β-sheet formation were observed. TEM (Fig. 1d) revealed a wide range (1–5 nm) of AβO diameters, with few protofibrils observed. These findings support the MDS results obtained at the start of incubation (0 h) in terms of the formation of AβOs. Within 1 h after the start of incubation, the Aβ_42_ monomers readily formed large, spherical AβOs ranging from 10 to 15 nm in size, and numerous protofibrils were observed (lengths of 50–80 nm). A TEM image at 3 h revealed the elongation of AβOs to form protofibrils, and significant amounts of large AβOs and protofibrils were observed at 6 h by TEM. A substantial decline in the MDS signal was observed during the time interval of 24–48 h after the start of incubation. TEM revealed the predominance of protofibrils and fibrils (over 120 nm in length) at 24 h, whereas AβOs were rarely observed, and the continuous maturation of protofibrils resulted in an increase in Aβ_42_ fibrils at 48 h. On the basis of these findings, the MDS sensitively and specifically detects oligomeric and protofibril forms of Aβ, permitting their quantification, whereas the ThT assay is not sensitive and was incapable of detecting increases in Aβ fibril levels, including diverse types of amyloid fibrils [34].

**Fig. 2:**
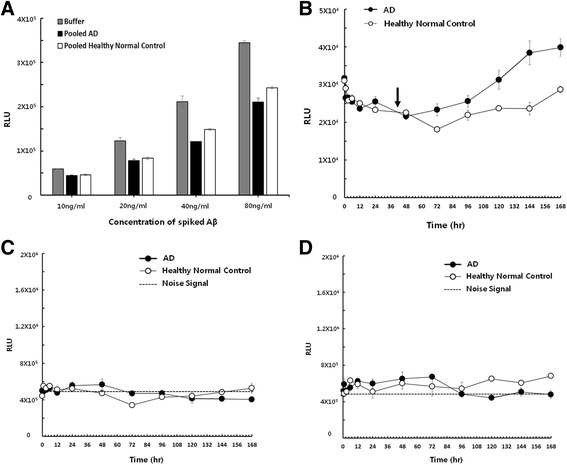
Changes of amyloid-β oligomers (AβOs) and amyloid-β (Aβ) monomers in pooled plasma of patients with Alzheimer’s disease (AD) and healthy normal control subjects. **a** After being spiked with different concentrations of Aβ_42_ in buffer vehicle, pooled AD plasma, and pooled healthy normal control plasma, oligomers were measured using a multimer detection system (MDS) at 0 h. **b** Pooled plasma samples were incubated at 37 °C after being spiked with 10 ng/ml Aβ_42_. Changes of Aβ_42_ levels at various time points were measured by MDS. Levels of Aβ_40_ (**c**) and Aβ_42_ (**d**) after being spiked with 10 ng/ml Aβ_42_ in pooled human plasma. Pooled plasma samples were incubated at 37 °C after 10 ng/ml Aβ_42_ were spiked. Data are mean ± SD. *RLU* Relative luminescence units. Arrow indicated no change in oligomer levels after an incubation period of 24 h

Plasma is cleared of Aβ through several intricate mechanisms of aggregation or sequestration [35–38]. Therefore, pooled samples of plasma from patients with AD or from normal control subjects were spiked with different concentrations of Aβ_42_ to compare differences in Aβ_42_ recovery (Fig. 2a and b). AβO levels were reduced in accordance with the Aβ_42_ concentrations used for spiking when compared with AβO levels in a solution spiked with buffer. Lower concentrations of spiked Aβ_42_ yielded smaller differences in the formation of AβOs in the pooled plasma of patients with AD compared with that of control subjects. For subsequent experiments, 10 ng/ml Aβ_42_ was chosen for spiking into plasma because this concentration yielded the smallest measurable difference in the recovery rate of Aβ when comparing plasma from patients with AD with that of normal control subjects. Eleven plasma samples from patients with AD and nine from elderly normal control subjects were separately pooled for each group. Pooled samples were incubated at 37 °C after spiking with Aβ_42_ (10 ng/ml), and the presence of oligomers was measured using MDS at various time points after the start of incubation. As shown in Fig. 2b, both groups exhibited a gradual decline in oligomer levels over 48 h of incubation; the two groups demonstrated similar levels over this time period. After 48 h of incubation, distinct dynamic changes were observed between the plasma from patients with AD and that of elderly normal control subjects. Larger increases in AβO levels were observed in the plasma from patients with AD after 48 h of incubation, and levels continually increased throughout the rest of the incubation period. In contrast, AβO levels in the plasma from elderly normal control subjects gradually decreased until 72 h after the start of incubation, then rebounded with a considerable increase until 144 h. The largest differences in AβO levels between plasma from patients with AD and plasma from elderly normal control subjects were observed after 144 h of incubation following spiking with Aβ. Changes in Aβ_40_ and Aβ_42_ levels after spiking with Aβ_42_ were also measured by performing 11A50/1E11-biotin ELISA and 12F4/1E11-biotin ELISA. Aβ_40_ and Aβ_42_ levels remained relatively unchanged over the incubation period (Fig. 2c and d), whereas significant increases in the oligomer forms, as measured by MDS, were observed in the plasma from patients with AD after 48 h of incubation but not in plasma from elderly normal control subjects. Additional experiments were then performed to confirm whether the differential changes in the Aβ forms were discernible in individual plasma samples.

**Fig. 3:**
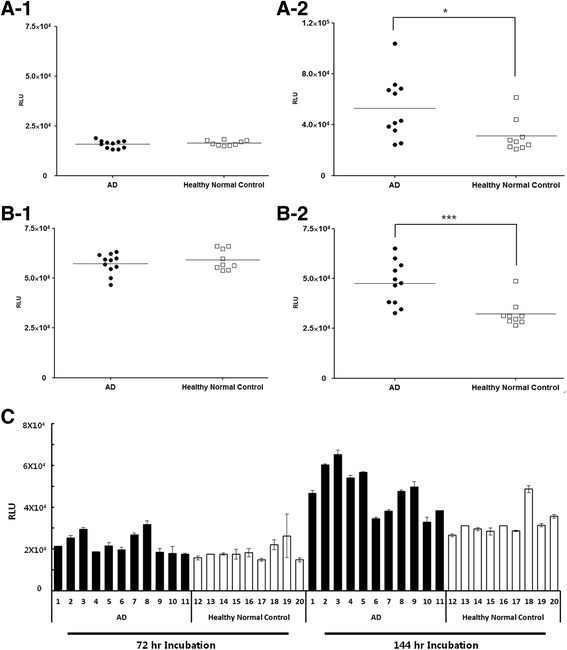
Effect of amyloid-β 1–42 peptide (Aβ_42_) spiking and incubation in individual subjects (Alzheimer’s disease [AD] and healthy normal control subjects). Each sample was incubated under the indicated conditions. To measure amyloid-β oligomers, a multimer detection system (MDS) assay was used. (**a1**) 0 ng/ml Aβ_42_ at 0 h, (**a2**) 0 ng/ml Aβ_42_ at 144 h, (**b1**) 10 ng/ml Aβ_42_ at 0 h, and (**b2**) 10 ng/ml Aβ_42_ at 144 h. (**c**) Dynamic changes of Aβ_42_ incubation in individual subjects at 72 h and 144 h. Each sample was incubated for 72 h and 144 h after being spiked with 10 ng/ml Aβ_42_ and measured by MDS. *p < 0.05; ** *p* < 0.01. *RLU* Relative luminescence units

Plasma samples corresponding to individuals included in the pooled groups (patients with AD and elderly normal control subjects) were spiked with Aβ_42_. Then, MDS was used to monitor changes in AβO levels during incubation from 0 to 144 h (Fig. 3a and b). Each sample was also tested in the absence of synthetic Aβ for comparison. As shown in Fig. 3b1, AβO levels in plasma samples from patients with AD and elderly normal control subjects overlapped without a significant difference (*p* = 0.6761) at 0 h, regardless of spiking. In contrast, after 144 h of incubation, distinct oligomer levels were detected in Aβ_42_-spiked samples from the AD and control groups (*p* < 0.01) on the basis of MDS measurements (Fig. 3b2). Plasma samples from the AD group also demonstrated higher AβO levels than normal control plasma, although this difference was significant but the *p* value was  < 0.05 (Fig. 3a2). Similar to the results of the pooled plasma experiment, Aβ dynamics were evident from 48 to 144 h in individual samples from both groups. As shown in Fig. 3c, substantial increases in oligomer levels were confirmed in the majority of plasma samples from patients with AD, whereas samples from normal control subjects demonstrated no significant increase. No increases in oligomer levels were observed with synthetic Aβ_42_ in buffer solution after an incubation time of 24 h.

### Differential dynamic changes in Aβ levels in plasma of patients with AD versus that of elderly normal control subjects

**Fig. 4:**
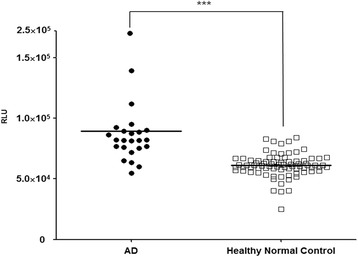
Effect of amyloid-β 1–42 peptide (Aβ_42_) spiking and incubation in large scale (Alzheimer’s disease [AD] and healthy normal control subjects). Each sample was incubated for 144 h after being spiked with 10 ng/ml Aβ_42_. To measure amyloid-β oligomers, a multimer detection system assay was used. ***p < 0.001 versus healthy normal control values. *RLU* Relative luminescence units

To evaluate and verify our findings on a larger scale, 24 plasma samples from clinically well-characterized cases of AD and 80 from elderly normal control subjects were examined after spiking with synthetic Aβ (10 ng/ml) and incubating for 144 h. The dynamic changes of oligomer formation were measured using MDS, and oligomer levels were different between the AD and control groups with a sensitivity of 83.33%, a specificity of 90.00%, an AUC of 0.8969, and a *p* value < 0.0001 (Fig. 4).

## Discussion

MDS was initially developed to detect prion disease. This method consists of a sandwich ELISA that exclusively detects oligomeric forms of antigens and relies on two different epitope-overlapping antibodies to capture and detect antigens by creating steric hindrance over a specific epitope [33, 39]. MDS was modified to detect AβOs for AD diagnosis using two epitope-overlapping Aβ antibodies specific for the N-terminus amino acids 1–18 of Aβ_42_.

We monitored the formation of AβO, protofibrils, and fibrils from synthetic Aβ_42_ in PBST buffer employing two methods: a ThT assay and MDS (Fig. 1). Aβ oligomerization was closely correlated between these two assays. When the time-course changes of synthetic Aβ in PBST buffer were monitored by MDS, ThT assay, and TEM, the MDS detected a continuous increase of Aβ_42_ oligomer levels from 0 to 24 h from the start of incubation, followed by a decline until 120 h. Conversely, an increase of fluorescent signal in the ThT assay was correlated with an increase of Aβ_42_ fibril formation, which was confirmed by TEM imaging (Fig. 1c and d). The MDS signal decreased at that time of fibrillization; thus, it would be reasonable to assume that MDS detected specifically oligomeric and protofibril forms of Aβ. During the early phase of incubation, MDS revealed decreasing AβO levels accompanied by the formation of protofibrils and fibrils, but the levels detected by the ThT assay were stable. Thus, MDS is able to detect AβOs during the early stage of Aβ oligomerization. Decreasing AβO levels, as measured by MDS, in conjunction with the formation of protofibrils and fibrils is potentially explained by two hypotheses. First, MDS has a higher sensitivity for oligomers than for protofibrils or fibrils. Second, the total number of oligomers decreases over time. After full oligomerization, the spiked Aβ converts to protofibrils, resulting in decreased MDS signals.

When synthetic Aβ_42_ is spiked into plasma, the plasma composition may dictate and interfere with Aβ oligomerization. Difficulties in detecting spiked synthetic Aβ_42_ via routine ELISA may be attributable to the presence of many different interfering factors in the plasma, which bind to the spiked synthetic Aβ_42_ and thus reduce its detection. These factors include naturally occurring Aβ autoantibodies, albumin, fibrinogen, immunoglobulin, ApoJ, ApoE, transthyretin, α_2_-macroglobulin, serum amyloid P component, plasminogen, and amylin [35–38, 40]. In addition, these molecules in bodily fluids could also inhibit Aβ fibrillization. Our hypothesis was that the composition of those components in blood from patients with AD would be different from that in healthy control subjects. If the same amount of Aβ were spiked into AD and control plasma samples, a different phenomenon would be observed between the two groups.

In this study, when equal amounts of synthetic Aβ_42_ were spiked into plasma, MDS signals for AβOs declined from 0 to 48 h in both groups (Fig. 2b), potentially due to AβO binding to interfering factors. Although binding affinities and Aβ epitopes likely vary among different binding factors, high concentrations in the plasma would likely result in the scavenging of spiked synthetic Aβ_42_. Binding of these factors to Aβ may naturally influence the normal functions and sequestration of Aβ, leading to clearance and reduced oligomerization potential in elderly normal persons [35, 37].

Forty-eight hours after spiking with Aβ_42_, AβO levels measured by MDS began to increase in the plasma from patients with AD but not in that of normal control subjects (Fig. 2b). Patients with AD may exhibit different binding profiles based on their plasma composition in the context of spiked synthetic Aβ_42_, which in turn may increase oligomerization potential and decrease sequestration capacity. Alternatively, the characteristics of endogenous plasma Aβ may differ between the two groups, permitting the dynamic changes of Aβ oligomerization to be detected.

It is challenging work to detect crude AβOs in plasma because the concentrations of Aβ in blood are very low. Furthermore, the concentrations of AβOs would be a subset of total Aβ in blood. The size of 4.5 kDa could be another reason why the MDS failed to measure the endogenous plasma Aβ from patients with AD. As shown in Fig. 3, the MDS did not discriminate between patients with AD and healthy normal control subjects without incubation conditions. Even though incubation of plasma samples without spiking external Aβ made slight differences between AD and normal control samples, the difference was not significant.

However, even at ultralow concentrations, the formation of AβO in the blood of patients with AD may be initiated via incubation with spiked synthetic Aβ_42_. The first 48 h of incubation represent a slow nucleation-dependent oligomerization phase during which ultralow concentrations of AβO nuclei are required to bind to spiked synthetic Aβ_42_. The period after 48 h and up to 144 h represents a rapid-growth phase for the formation of oligomers, protofibrils and fibrils, surpassing the critical detection limit [34, 41–43]. As previously mentioned, the interfering factors may be saturated with spiked Aβ; hence, they may not affect to MDS signal even after 48 h of incubation. In addition, the binding interactions between Aβ and another Aβ may be stronger than other interfering factors for oligomerization. In previous studies, by using protein misfolding cyclic amplification technology, researchers were able to differentiate AβO levels by catalyzing the misfolding and amplification of Aβ aggregates by spiking Aβ_42_ into the CSF of patients with AD and control individuals [44]. However, spiking Aβ into the plasma to differentiate AβO has not previously been published.

We observed the phenomenon that MDS signals of control subjects were still stable while we spiked the same large amount of synthetic peptide into both AD and normal plasma samples, even after the identical incubation step. Currently, we do not know the exact cause of the phenomenon. Oligomerization of Aβ could be influenced by potential factors in plasma of patients with AD but not plasma samples from normal control. The concentrations of these potential factors could be different in the disease state, but they may not be present in the normal state.

We detected differential AβO dynamic changes in the blood of patients with AD and normal control subjects, but a direct correlation between blood and brain pathology remains uncharacterized. The properties of Aβ plaques in the brain may differ from those in the blood because Aβ in the blood also originates from amyloid precursor protein metabolism in skeletal muscle, organs, skin, and peripheral cells [45, 46]. However, on the basis of previous reports, Aβ peptides cross the blood-brain barrier, resulting in elevated Aβ levels in the CSF and plasma during intracerebroventricular injection of synthetic Aβ_42_ monomers into normal imprinting control region mice [47, 48]. It will be interesting to identify the correlation between AβO concentrations in the plasma and amyloid plaque deposition in the brains of patients with AD.

## Conclusions

Spiked synthetic Aβ_42_ induced differential dynamic changes in AβO levels in the plasma of patients with AD compared with that of normal control subjects, as detected by MDS. These observations appear to support our hypothesis that the plasma composition and/or characteristics of endogenous Aβ in patients with AD versus normal healthy persons are different. To our knowledge, there have been no published reports involving the spiking of Aβ into plasma. The characterization of differential Aβ oligomerization dynamic changes may contribute to the development of blood-based biomarkers for AD. However, further studies are required to elucidate the mechanisms underlying the formation of AβOs. Longitudinal studies undertaken during the predementia stage of AD should also be carried to assess clinical applications for the early detection and monitoring of this disease.

## Abbreviations

Aβ: Amyloid-β
Aβ_42_: Amyloid-β 1–42 peptide
AβO: Amyloid-β oligomer
AD: Alzheimer’s disease
Apo: Apolipoprotein
CDR-SOB: Clinical Dementia Rating Sum of Boxes
CSF: Cerebrospinal fluid
ELISA: Enzyme-linked immunosorbent assay
FDG: ^18^F-fluorodeoxyglucose
HRP: Horseradish peroxidase
IL: Interleukin
MDS: Multimer detection system
miR: MicroRNA
MMSE: Mini Mental State Examination
PBST: Phosphate-buffered saline containing Tween 20
PET: Positron emission tomography
PiB: ^11^C-Pittsburgh compound B
p-Tau: Phosphorylated tau protein
RFU: Relative fluorescence unit
RLU: Relative luminescence unit
RT: Room temperature
sCD40: Soluble CD40
sCD40L: Soluble CD40 ligand
SUVR: Standardized uptake value ratio
TBST: Tris-buffered saline containing Tween 20
TEM: Transmission electron microscopy
ThT: Thioflavin T
t-Tau: Total tau protein
